# Applied Pathology

**Published:** 1914-06

**Authors:** 


**Applied Pathology.** By JULIUS M. BERNSTEIN, M.B., D.P.H. Pp. xvi, 395. London : University of London Press. [1913.] 10s. 6d. net.

Intended as a survey of the applications of clinical research to practical medicine for busy practitioners and senior students, this book fulfils its object. All technique is omitted save that relating to the collection of material.

The introduction discusses the scope of the clinical pathologist's work and the dangers of inadequate knowledge of clinical medicine and of the patients on the part of pathologists. This draws attention to the separation of the clinician and pathologist so common at present, a state of affairs which brings discredit on much that would otherwise be esteemed. The laboratory tests and products are only links in the chains which curb disease, these must be welded with the results of the clinical investigations if satisfactory results are to be expected. The pathologist and clinician should therefore consult together.

Dr. Bernstein favours relieving the directors of laboratories of the routine work, a very pleasant and tempting proposal. Laboratory attendants are experts at this work, but processes have to be modified often or important points would be missed. Specialised knowledge is required for this, therefore personal attention to all work is necessary. Routine investigations have necessarily to be left to assistants in "trade laboratories," hence much distrust in this work. Pathologists are often asked why they do not do work quickly at a low figure. An autogenous vaccine, it is said, can be obtained in twenty-four hours from x—— for a couple of shillings or thereabouts, or perhaps the outfit and materials for a Wassermann's test. The reply is, a pathologist does not compete with this work any more than the consultant with an open surgery. In both cases it is likely that where shillings are paid instead of guineas a different class of work is done.

The author shows that most stock vaccines are useless. A vaccine prepared in two days or less must be risky. The organisms cannot be grown, isolated and identified in the time; a shrewd guess may be made, but the result is possibly unsatisfactory. The futility of mutilated Wassermann's reactions is not shown. Only careful selection and titration of all reagents necessitating numerous controls can eliminate errors which render the results valueless. Even if short cuts were reliable, they do not give the degree of fixation of complement necessary for estimating the effect of salvarsan injections. The effect of salvarsan is not constant, one injection may accomplish what several do not in another case. Sometimes the fixation of complement gradually lessens after each injection; the treatment must be continued therefore, but the progress is shown.

Most of the illustrations are good, but figure 9 on page 6 is poor. The book is well proportioned and easy to read. It would take long to show an appreciation of even a portion of what ought to be praised. The portions inviting controversy have therefore been selected instead.

